# Coracoid osteotomy in anterior fracture-dislocation with concomitant bony Bankart: a way to safely retrieve the humeral head and provide instant stability (acute Latarjet)

**DOI:** 10.1016/j.xrrt.2021.10.007

**Published:** 2021-12-03

**Authors:** Merel Pape, Leanne Stephanie Blaas, Jian Zhang Yuan, Jacobus A. de Priester, Ansel R. Bruinenberg, Nikki Buijs, Robert Jan Derksen

**Affiliations:** Department of Traumasurgery, Zaandam Medical Center, Zaandam, the Netherlands

**Keywords:** Latarjet, Coracoid osteotomy, Proximal humerus fracture dislocation, Bony Bankart

Anterior glenohumeral fracture-dislocations are generally perceived as challenging to treat, especially when the injury also encompasses a (bony) Bankart lesion.^14^^,^^17^^,^^29^^,^^31^ These complex, multifaceted injuries are known for their poor functional outcomes.^31^ Therefore, optimal initial treatment has to include addressing the proximal humerus fracture as well as restoring joint stability to provide the best possible foundation for functional recovery.^3^^,^^29^ Several complicating factors surround both components of this “terrible dyad”. First, with regard to the proximal humerus fracture-dislocation, safe retrieval of the humeral head from the deltopectoral axillary groove without (further) injuring the already compromised blood supply is challenging. When the humeral head forcefully enters the anterior axillary deltopectoral groove during the trauma, it often injures the brachial plexus.^13^ Second, with regard to the bony Bankart lesion, the often accompanying instability of the glenohumeral joint after reduction and fixation of the humeral head proposes a considerable problem.^3^^,^^14^ For both the safe and least damaging retrieval of the humeral head as well as to achieve adequate glenohumeral stability in the acute setting, we propose a coracoid osteotomy as a “double-edged sword”.^2^^,^^18^^,^^26^ Especially in young, muscular patients, safe access to and soft-tissue-friendly retrieval of the humeral head is both challenging and of vital importance to achieve a reasonable prognosis.^17^ In elderly patients, the choice would readily be to perform a fracture reverse shoulder arthroplasty in these cases.^1^^,^^5^^,^^11^^,^^17^^,^^19^^,^^28^ However, in young, active patients, we are prompted to perform a humeral head-preserving reconstruction.

To the best of our knowledge, no previous manuscript describes a coracoid osteotomy in combination with an acute Latarjet procedure in the treatment of a proximal humerus fracture-dislocation with a concomitant bony Bankart lesion. Therefore, we describe a case in which a coracoid osteotomy was performed to provide safe access to the humeral head, using the osteotomized coracoid tip to perform an acute Latarjet procedure to provide glenohumeral stability.

## Case description

We describe the case of a 28-year-old male who was admitted to the emergency department after a motor vehicle accident, in which he crashed with his motorcycle. He fell on his outstretched right arm under and behind his torso, forcefully externally rotating and anteriorly displacing the humeral head. Primary survey yielded no life-threatening injuries. His main complaint was a painful, dysfunctional right shoulder. On physical examination, the shoulder was swollen; however, no hematoma or dislocation of the humeral head was clinically distinguishable. There was no neurovascular deficit. On palpation, the shoulder was markedly painful, and there was a profound restricted range of motion.

**Figure 1:**
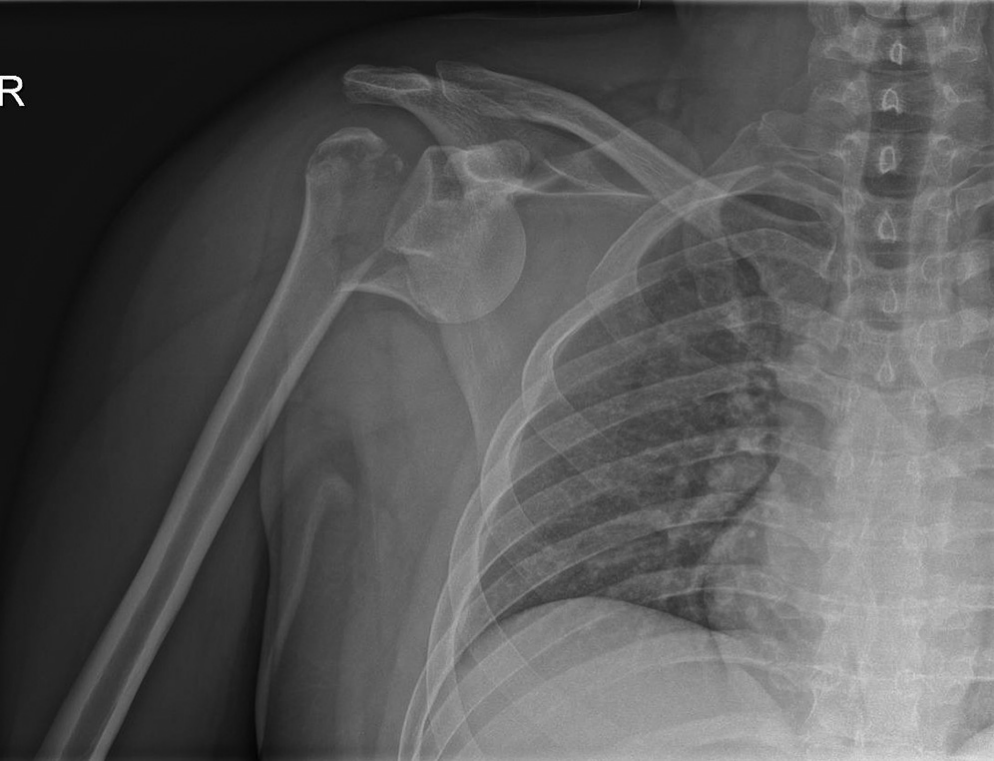
Radiograph of the preoperative shoulder showing a comminuted fracture-dislocation of the proximal humerus on the *right* side.

**Figure 2:**
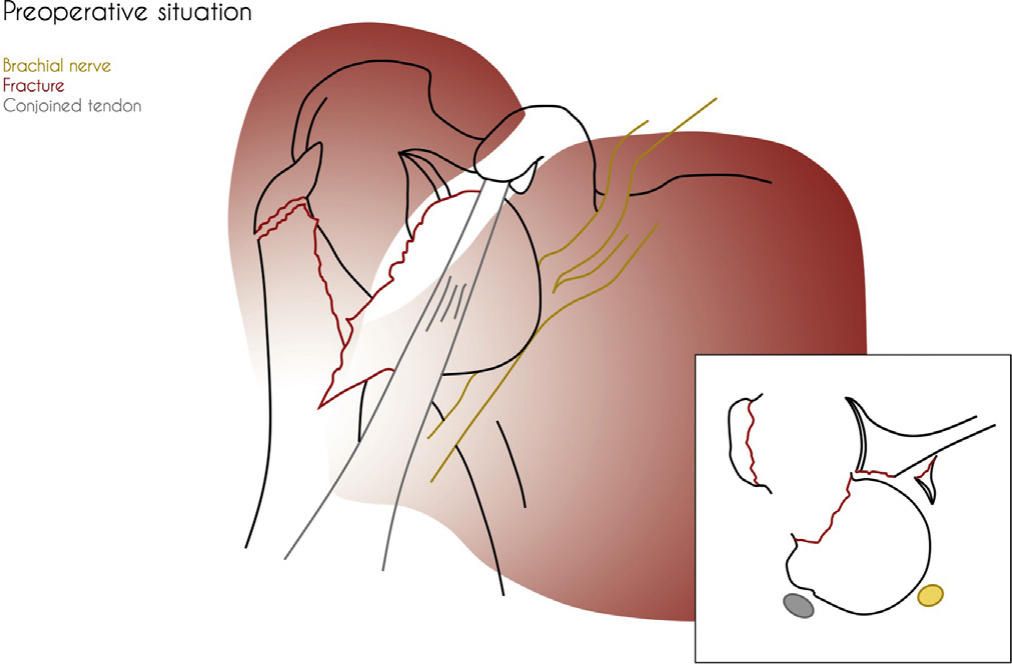
Preoperative situation showing a dislocated humeral head and fractured anterior glenoid rim (bony Bankart). The glenoid fragment is located posterior and caudally with respect to the humeral head.

His past medical history was unremarkable, only revealing an open appendectomy. Plain radiographs showed a comminuted fracture-dislocation of the proximal humerus on the right side (Figs. 1 and 2). The humeral head was dislocated medially and anteriorly, and concomitantly, the anterior glenoid rim was fractured and displaced (bony Bankart). The glenoid fragment was located posterior and caudally with respect to the humeral head. Computed tomography imaging of the brain, cervical spine, and thorax showed no other injuries.

The patient was admitted to the surgical ward and was prepared for open surgery to be performed the next day.

## Procedure

**Figure 3:**
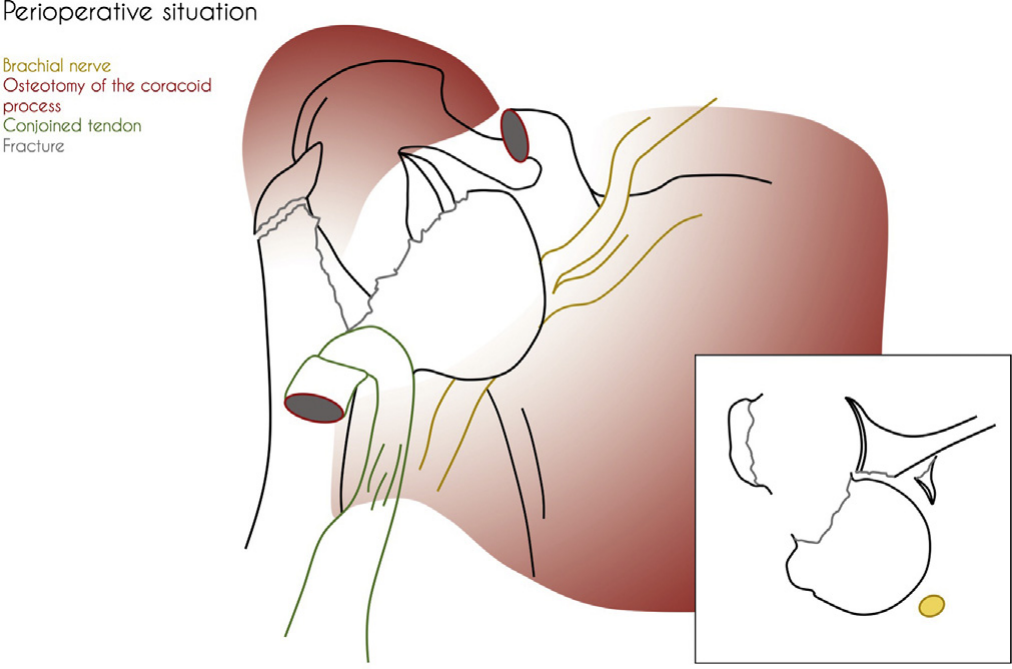
Perioperative situation showing the anatomical status of the dissected area.

**Figure 4:**
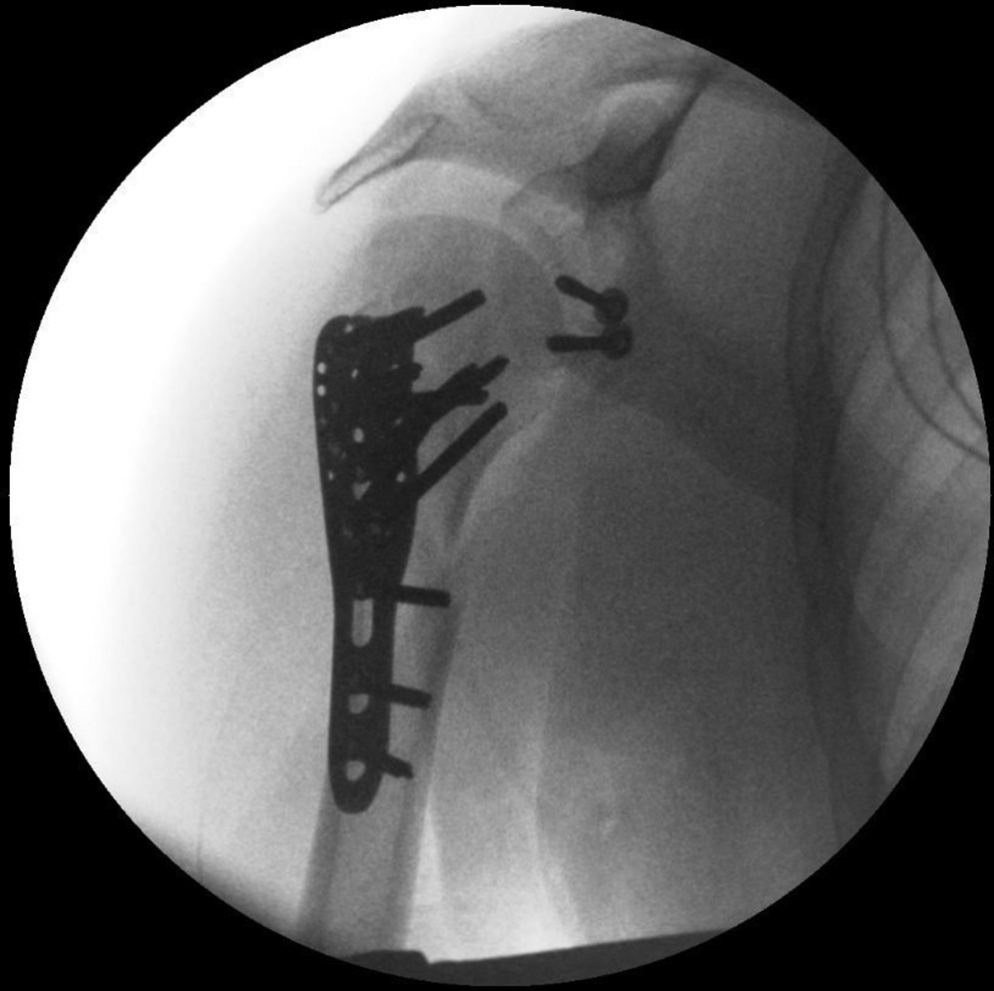
Perioperative fluoroscopy images showing the definitive osteosynthesis; a locking compression plate and two lag screws.

**Figure 5:**
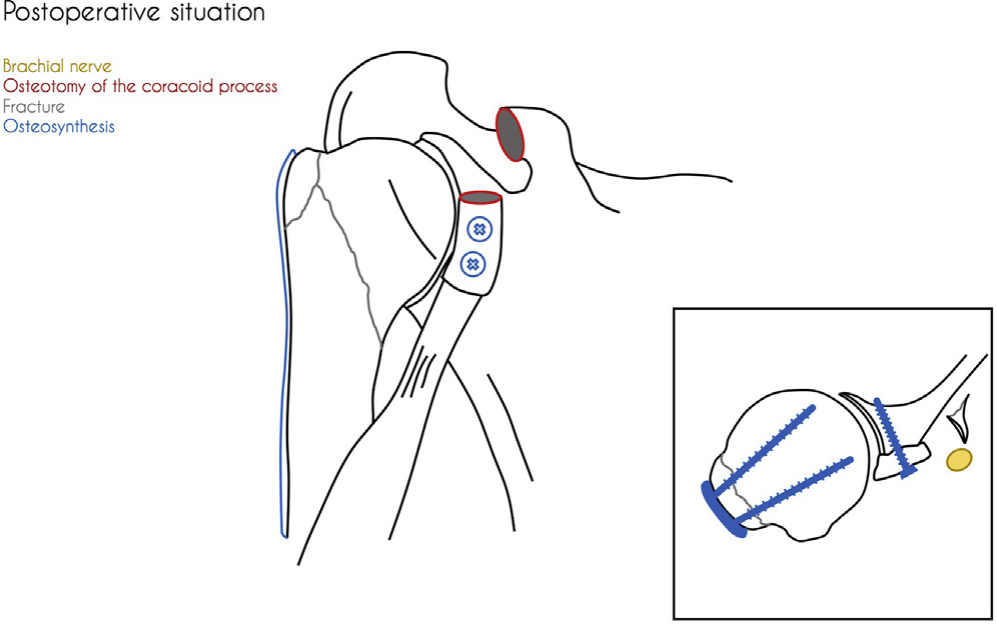
Postoperative situation showing the locking compression plate and screws in the coracoid process.

The patient received general anesthesia and was placed in the beach chair position. The shoulder was prepared and draped in the usual sterile fashion. A standard deltopectoral approach was performed. The humeral head was not safely retrievable from the anterior deltopectoral groove because of limited access caused by well-developed deltoid and pectoral muscles. An osteotomy of the coracoid process was performed to provide access to the dislocated humeral head, enabling retrieval of the humeral head without (further) damaging the brachial plexus. The humeral head was reduced together with the greater tubercle to the shaft and fixed with a locking compression plate (PHILOS; DePuy Synthes, Raynham, MA, USA). After having performed open reduction and internal fixation of the proximal humerus, it was noted that the humeral head failed to remain in its anatomical position because of the substantial bony Bankart lesion causing glenohumeral instability. To achieve stability without further compromising the brachial plexus (The anterior glenoid fragment was dislocated toward the brachial plexus.), an acute Latarjet procedure was performed with the already osteotomized coracoid tip (Fig. 3). The coracoid process was transferred together with its attached tendons (short head of biceps and coracobrachialis) through the subscapularis tendon and fixed anteriorly to the glenoid fossa at the avulsion site with two lag screws (Figs. 4 and 5). After rinsing the wound, the wound was closed in layers with uninterrupted resorbable sutures for the deltopectoral fascia, the superficial subcutaneous fascia, and skin.

## Follow-up

**Figure 6:**
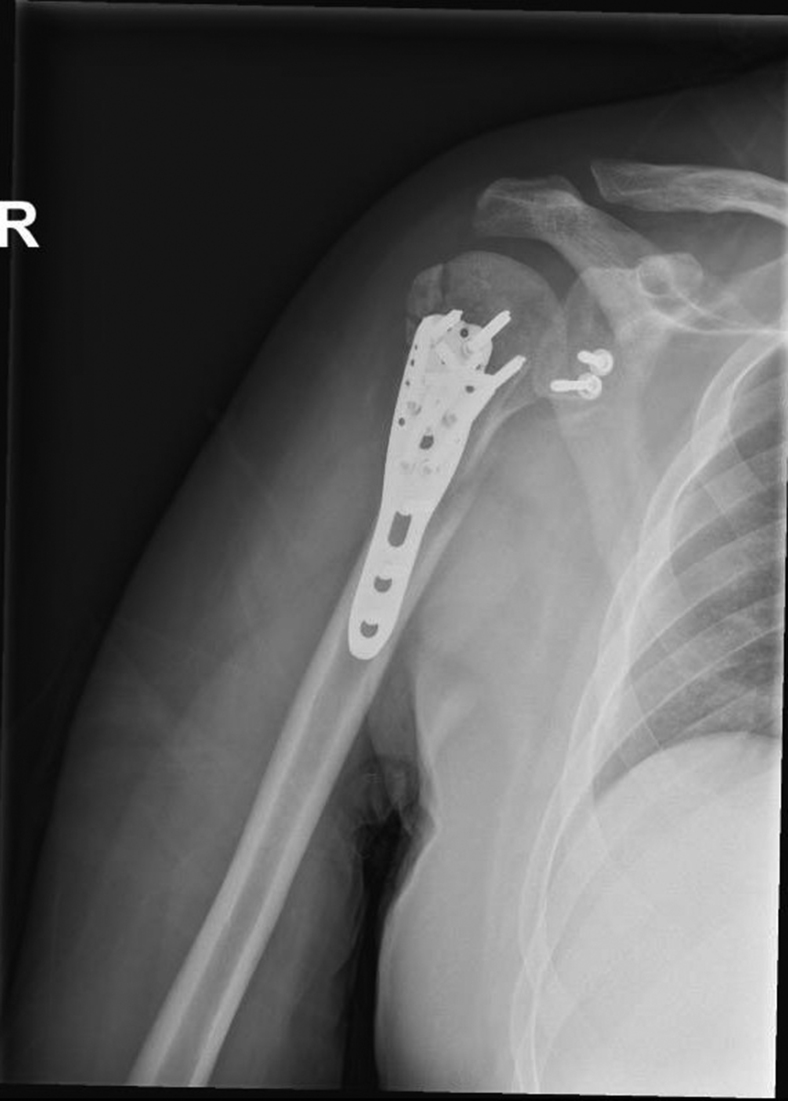
Postoperative lateral radiograph after 6 weeks, showing an adequate position of the humeral head, centrally opposing the glenoid.

Postoperatively, the patient returned to the surgical ward for observation and pain management. After 5 days, the patient was discharged from the hospital. He was instructed to wear a Gilchrist immobilizer (Actimove, Stockholm, Sweden) for four weeks. Moreover, he was allowed to take the immobilizer off to perform circumduction exercises three times a day. After 4 weeks, passive motion up to 90 degrees forward flexion and abduction was allowed. At six weeks postoperatively, he was seen at the outpatient clinic. The wound was healed, and no signs of infection were present. Conventional radiography showed an adequate position of the humeral head, centrally opposing the glenoid (Fig. 6). At this time, he was allowed to actively move the shoulder up to 90 degrees forward flexion and abduction; however, he was also advised to avoid external rotation. To further guide functional recovery, he was referred to a specialized shoulder physiotherapist. The first consultations were focused on reaching a painless abduction up to 90 degrees and full range of motion of the nearby joints (the elbow and wrist).

**Figure 7:**
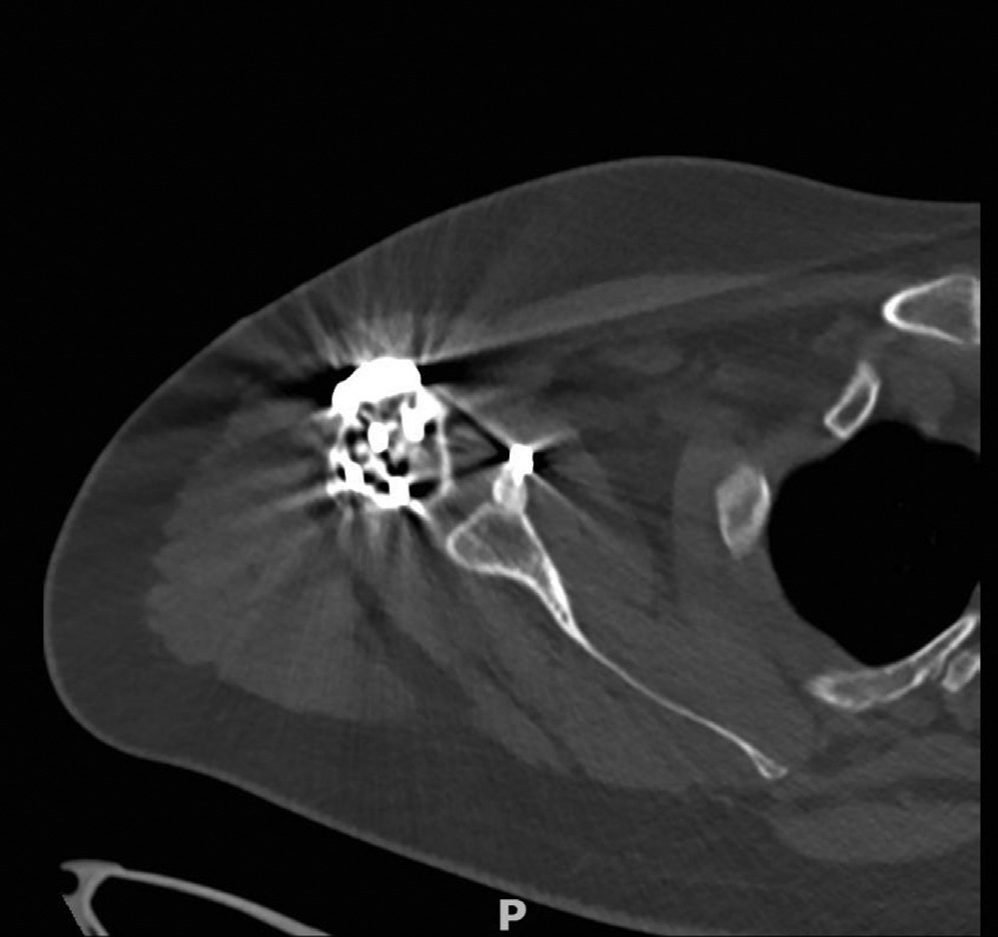
Postoperative axial computed tomography scan after 8 months, showing complete union of the proximal humerus fracture and the coracoid osteotomy.

Computed tomography imaging at 8 months postoperatively showed complete union of the proximal humerus fracture and Latarjet (Fig. 7). However, it was seen that the tip of the plate impinged subacromially, for which he was scheduled to undergo plate removal. Ultrasound imaging furthermore revealed intact rotator cuff tendons.

**Figure 8:**
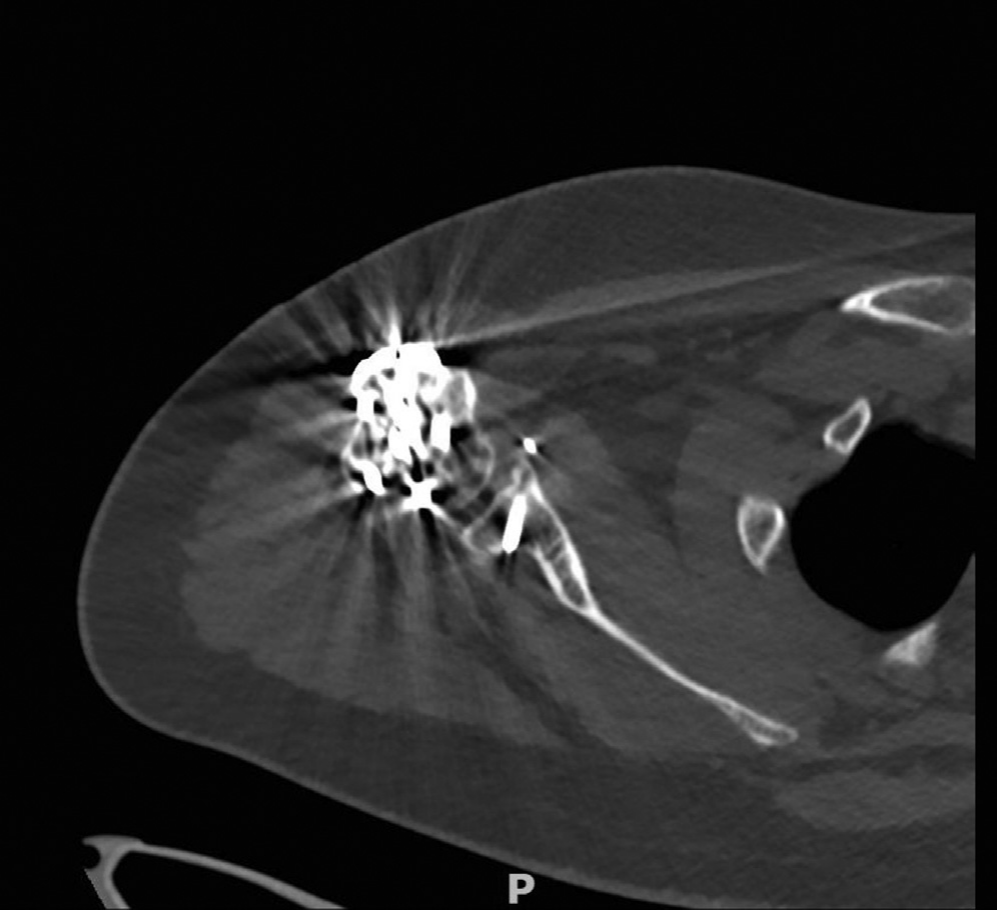
Another slide of the postoperative axial computed tomography scan after 8 months, showing complete union of the coracoid transfer and proximal humerus fracture.

**Figure 9:**
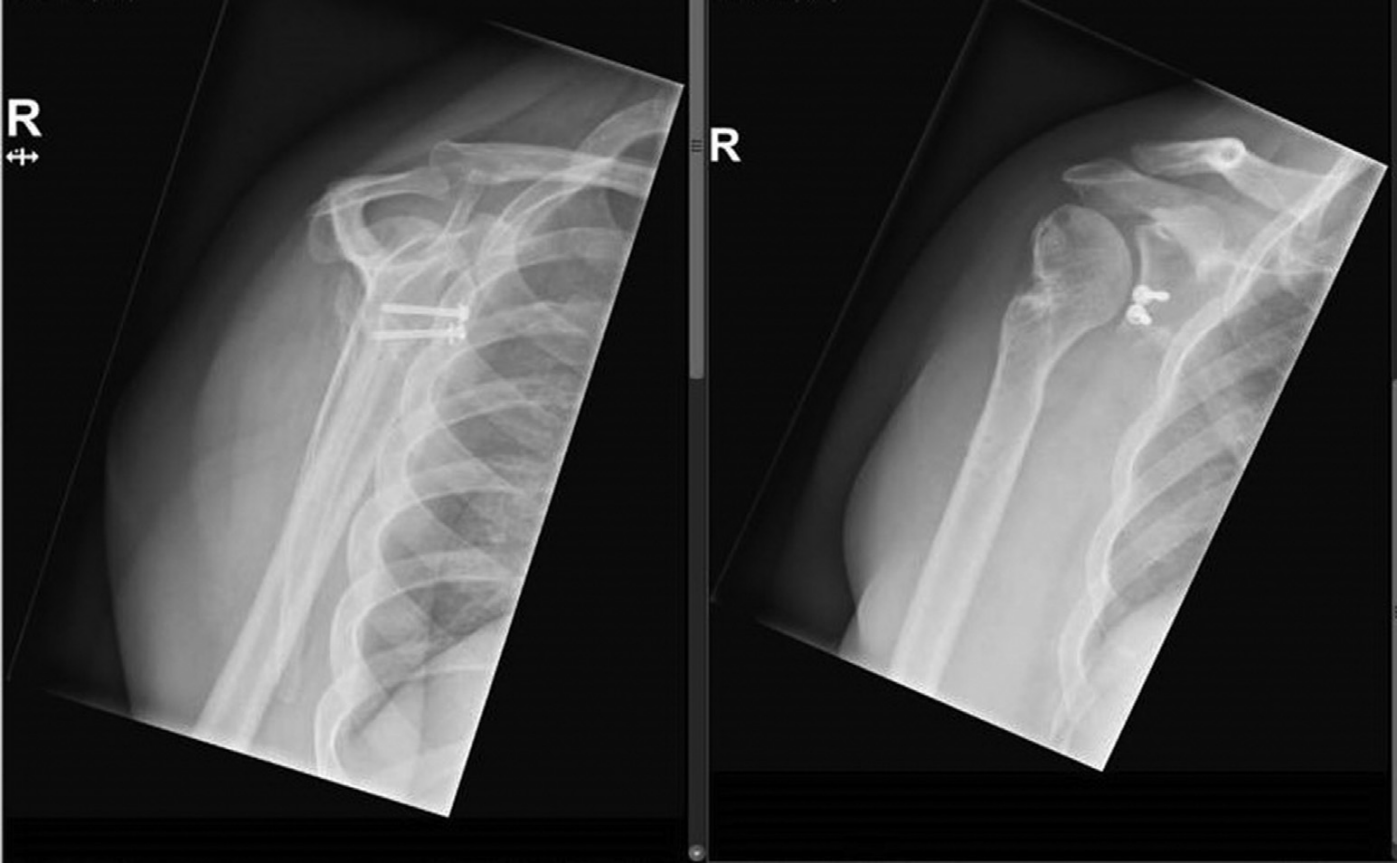
Postoperative radiographs after the removal of the humeral plate and screw fixation, showing an adequate position of the humeral head and the two lag screws of the Latarjet procedure.

**Figure 10:**
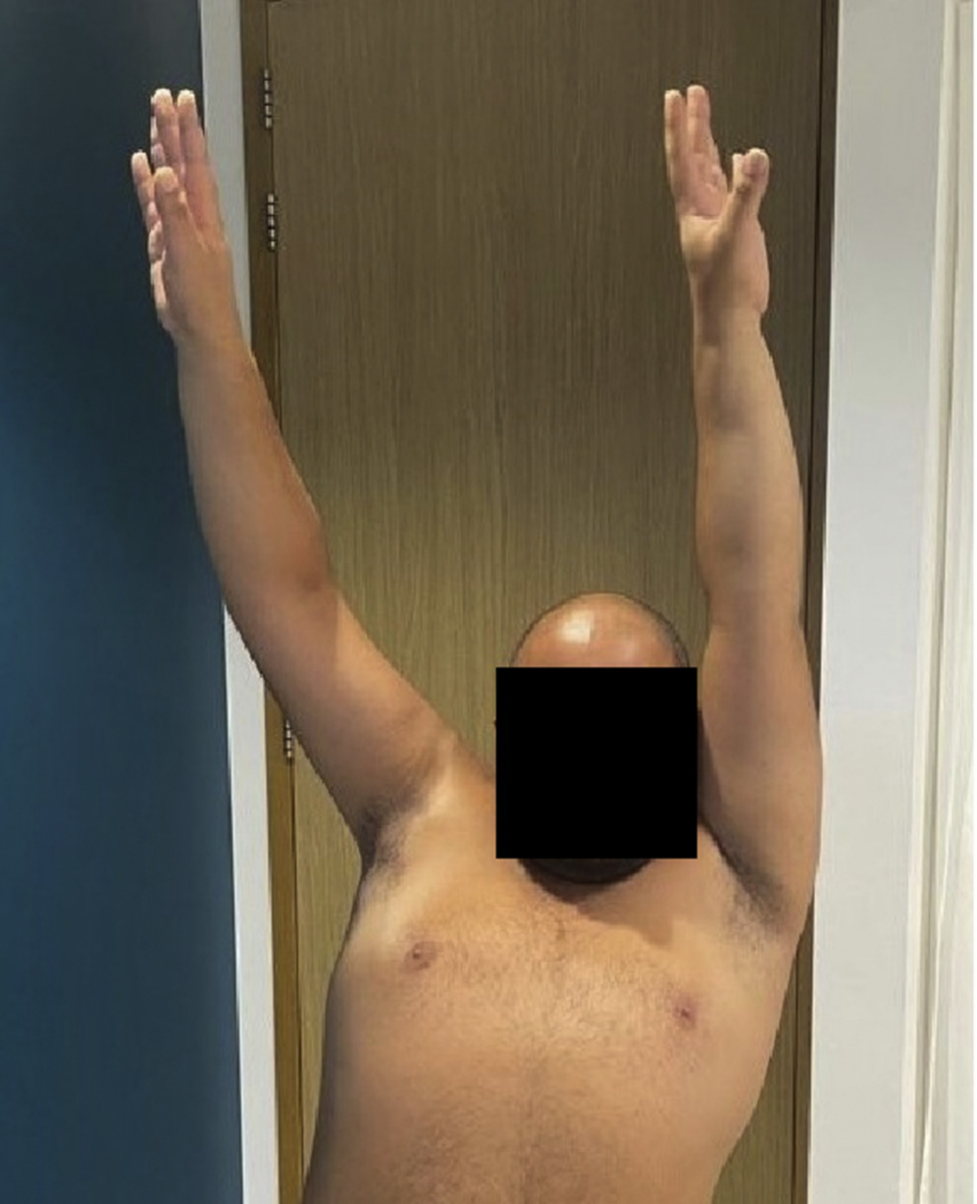
A photograph taken at the outpatient clinic, showing the level of abduction after one year of follow-up.

**Figure 11:**
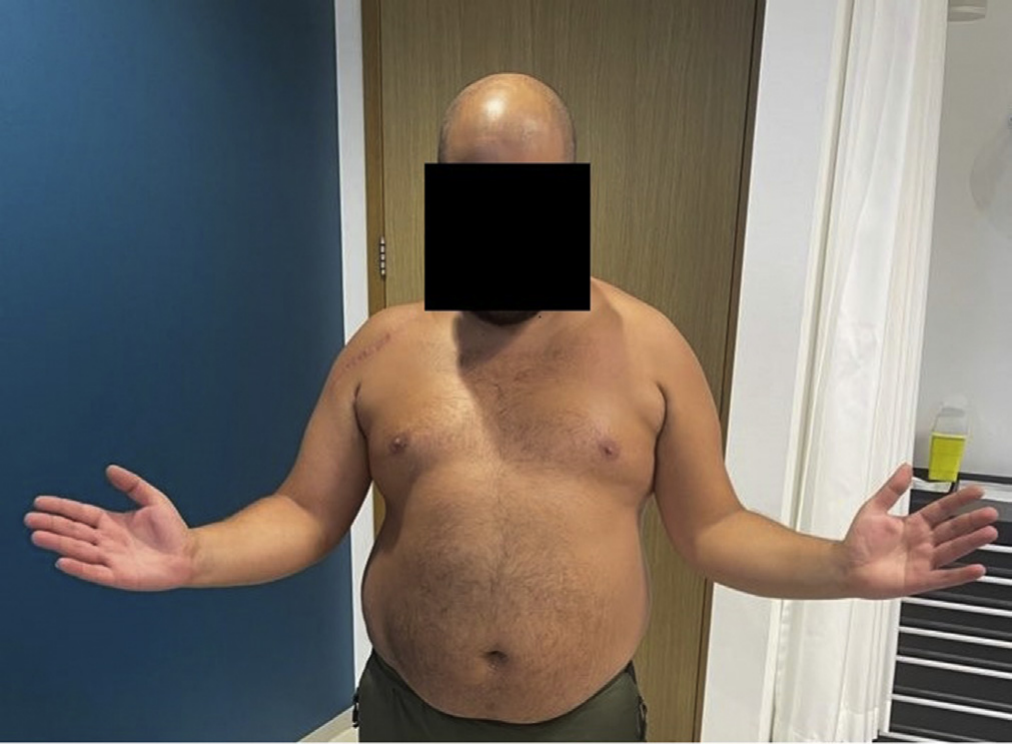
A photograph taken at the outpatient clinic, showing the level of external rotation after one year of follow-up.

At 9 months, he was scheduled for a second operation where the plate and screws of the humeral plate were removed because of the aforementioned reasons (Fig. 8). At his latest outpatient clinic visit (one year after the initial trauma and surgery), he was able to reach an active forward flexion of 150 degrees, 130 degrees of abduction, and both 90 degrees internal and external rotation (Figure 9, Figure 10, Figure 11). Using the visual analog scale pain score, he experienced a low mean pain score of 1, and he stated his worst pain score as 2. Common daily activities, such as showering, getting dressed, and doing grocery shopping, were performed painlessly. The Disabilities of the Arm, Shoulder and Hand (DASH) score at one year postoperatively for his right arm was 11 points (range 0-100 with higher scores indicating a significant impairment in hand, arm, and shoulder function)^16^. The Oxford Shoulder Score for this patient was 41 points (range 0-48), with 48 being the highest score achievable.^8^ This Oxford Shoulder Score suggests a satisfactory joint function of his right shoulder function. Finally, we took the Constant Shoulder Score for both his shoulders, in which he achieved 78 points and 100 points for his right and left shoulder, respectively. A difference in scores of 22 points between his operated and unaffected shoulder indicates a fair functional level.^6^^,^^7^ Guided shoulder physiotherapy showed that his ROM has further improved.

## Discussion

The Latarjet procedure, first described in 1954, is a well-known and successful technique to repair a glenoid rim defect using the coracoid process as a bone graft, mostly performed in patients suffering from recurrent anterior shoulder dislocations and chronic glenohumeral instability.^10^^,^^22^ In contrast to the previously described indications, we executed this procedure in a proximal humerus fracture-dislocation in the acute setting. These injuries create a significant risk of developing avascular necrosis (AVN) of the humeral head because the delicate capsular vessels are often (partially) torn.^15^^,^^25^^,^^29^^,^^30^ Retrieving the humeral head in a manner that preserves the residual arterial supply is key to optimize the chance of preventing secondary AVN. Furthermore, the humeral head lies adjacent to the brachial plexus, and retrieving the humeral head in these complex injuries can easily cause further damage to the nerves. In 75%-89% of cases, stretching the brachial plexus causes transient neuropraxia, and in 2.7%, a (partially) torn brachial plexus is seen in the mechanism of injury described as in our case.^4^^,^^12^^,^^13^ A coracoid osteotomy is a known maneuver to enhance exposure for structures in the anterior deltopectoral groove (eg, neurovascular structures, such as the brachial plexus).^2^^,^^18^^,^^20^^,^^23^^,^^26^ Further damage to the delicate arterial supply of the humeral head, resulting in AVN, is prevented in this manner.^2^^,^^18^^,^^26^

Finally, a coracoid osteotomy provides the possibility to restore glenohumeral stability. Often in these injuries, the bony fragment and torn labrum are pushed away by the humeral head and are forced toward the brachial plexus. Retrieval of these fragments is both hazardous with regard to the adjacent nerve structures as well as insufficient to restore glenohumeral stability. Generally, the fractures encompass merely small bony fragments and a damaged labrum. Acute Bankart repair with anchors and capsuloplasty was not deemed feasible in the acute setting because the capsule is irregularly torn and difficult to distinguish because of hematoma and displacement.^9^^,^^24^ Furthermore, the Bankart repair is associated with a higher recurrence rate than the Latarjet procedure.^14^^,^^21^^,^^27^

The mechanism of the Latarjet procedure is not fully understood; however, it has been described as a combination of three possible effects.^9^^,^^22^ The first effect is the dynamic “sling” of the conjoint tendon, in which the lower part of the subscapularis muscle and the capsule stabilizes the shoulder during abduction and external rotation.^22^ The second stabilizing mechanism, the repair of the capsulolabral complex to the bone or the coracoacromial ligament, accounts for additional stability.^9^^,^^22^ However, owing to an irregularly torn capsule and hematoma, we were not able to perform a capsuloplasty in the acute setting; hence, this stabilizing mechanism is missing in our patient. The final stabilizing effect is realized by reconstructing the glenoid concavity by using the coracoid process, which contributes to the glenohumeral stability.^22^

Although the Latarjet procedure has shown good clinical results and outcomes, it has been associated with a postoperative complication rate varying between 15% and 25%.^9^^,^^22^ The most commonly reported complications are infection, frozen shoulder, nonunion of the coracoid graft, bone resorption, recurrence of instability, and neurological complications.^9^^,^^22^ In this relatively short follow-up period, our patient developed no complications with regard to the Latarjet procedure.

**Figure 12:**
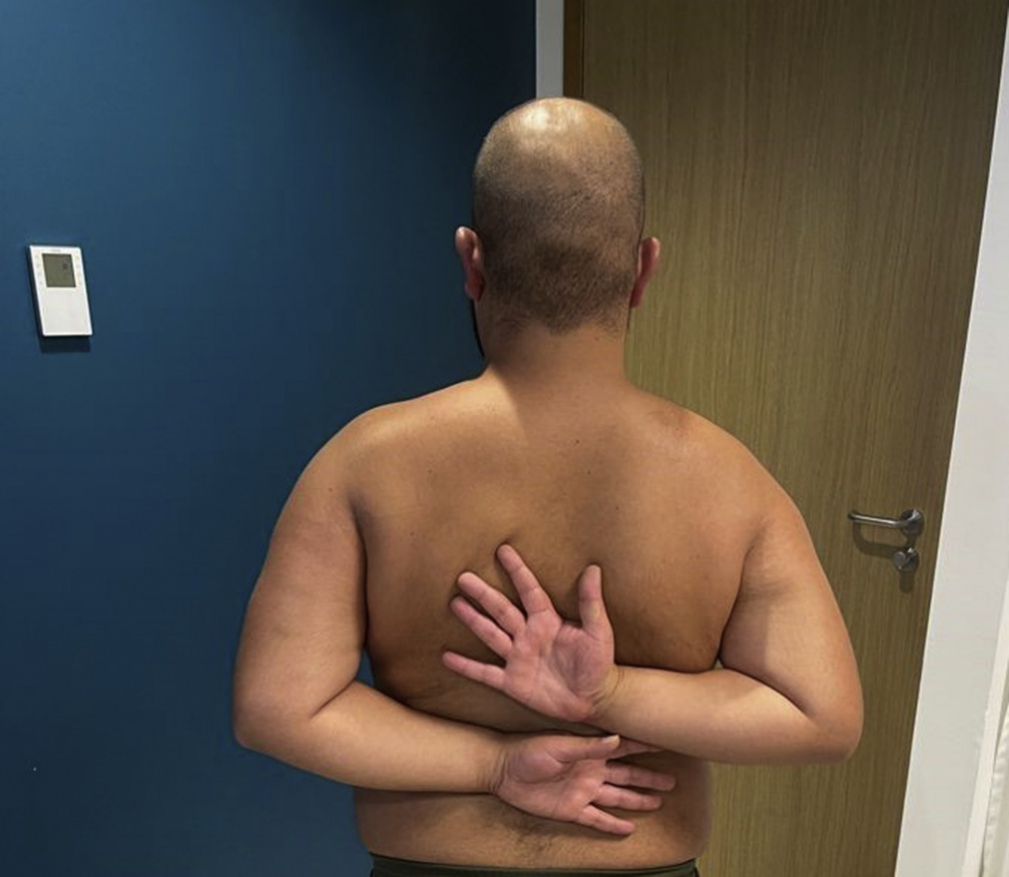
A photograph taken at the outpatient clinic, showing the level of internal rotation after one year of follow-up.

To our knowledge, this is the first case report to describe an acute Latarjet procedure in the treatment of a bony Bankart lesion and a proximal humerus fracture in a young male patient. Our case demonstrated an example of the benefit of tailored, intraoperative decision-making in a complex, multifaceted injury. Owing to subacromial impingement for which plate removal has been scheduled, his functional outcome is moderate after eight months of follow-up (DASH score of 39). After one year of postoperative follow-up, we saw satisfactory results in range of motion and his right shoulder function (DASH score of 11) (Figure 9, Figure 10, Figure 11, Figure 12). Obviously, this concerns just one case and limited follow-up, so careful interpretation is warranted. For example, there still is the possibility of AVN development. However, the benefit of the coracoid osteotomy and transposition in this case is undeniable in our opinion. By reporting this case, we want to convey the promising possibilities of a coracoid osteotomy and subsequent acute Latarjet in the management of these challenging injuries.

## Conclusion

We described a case of an anterior glenohumeral fracture-dislocation in which a coracoid osteotomy and an acute Latarjet were performed to both safely retrieve the humeral head and restore glenohumeral stability. We found this to be a suitable method to minimize further damage of the humeral head blood supply and to prevent damaging the brachial plexus. In conclusion, we feel that in anterior glenohumeral fracture-dislocations also comprising a bony Bankart, coracoid osteotomy could be an effective “double-edged” sword to safely retrieve the humeral head and restore joint stability.

## Disclaimers

Funding: No funding was disclosed by the authors.

Conflicts of interest: The authors, their immediate families, and any research foundation with which they are affiliated have not received any financial payments or other benefits from any commercial entity related to the subject of this article.
