# Supplementary material for: Coracoid osteotomy in anterior fracture-dislocation with concomitant bony Bankart: a way to safely retrieve the humeral head and provide instant stability (acute Latarjet)
Source: JSES Rev Rep Tech. 2021 Dec 3;2(1):40–5. doi: 10.1016/j.xrrt.2021.10.007 (PMC10426480; doi:10.1016/j.xrrt.2021.10.007)
Supplement: Supplementary Material [file mmc1.docx]

**Legends page of figures**

**Figure 1** Radiograph of the preoperative situation showing a comminuted fracture-dislocation of the proximal humerus on the right side.

**Figure 2** Preoperative situation showing a dislocated humeral head and fractured anterior glenoid rim (bony Bankart). The glenoid fragment is located posterior- and caudally respective to the humeral head.

**Figure 3** Perioperative situation showing the anatomical status of the dissected area.

**Figure 4** Perioperative fluoroscopy images showing the definitive osteosynthesis; a locking compression plate and two lag screws.

**Figure 5** Postoperative situation showing the anatomical status and definitive osteosynthesis; a locking compression plate and two lag screws.

**Figure 6** Postoperative lateral radiograph after 6 weeks, showing an adequate position of the humeral head, centrally opposing the glenoid.

**Figure 7** Postoperative axial CT-scan after 8 months, showing complete union of the proximal humerus fracture and the coracoid osteotomy.

**Figure 8** Another slide of the postoperative axial CT-scan after 8 months, showing complete union of the coracoid transfer and proximal humerus fracture.

**Figure 9** Postoperative radiographs after the removal of the humeral plate and screw fixation, showing an adequate position of the humeral head and the two lag screws of the Latarjet procedure.

**Figure 10** A photograph taken at the outpatient clinic, showing the level of abduction after one year of follow up.

**Figure 11** A photograph taken at the outpatient clinic, showing the level of external rotation after one year of follow up.

**Figure 12** A photograph taken at the outpatient clinic, showing the level of internal rotation after one year of follow up.
